# 

**Published:** 1909-01-15

**Authors:** 


### SUBJECT INDEX TO VOLUME LXIII.

EVENT AND COMMENT.

Alveolar Abscess, 1.

Business Building, 552.

Cast Inlay Patients, 110.

Dental Books, 109.

Dental Caries, 2.

Dental Journalism, 56.

Dental Progress in 1909, 606.

Dental Work for School Children, 605.

Dentist's Magazine, 227.

Fletcherism, 173.

Fletcher's Kindergarten, etc., 174.

Fifth International Congress, 228, 498.

Honors to Our Great Men, 607.

Increasing Our Literary Output, 389.

Institute of Dental Pedagogics, 55.

Loving Cup to the Miller Dental Club, 551.

Miller Dental Club, 497.

Plethoric Programmes, 443.

Proposed English Anesthetic Law, 335.

Silicious Cements, 281.

Dr. Taggart and the Profession, 111.

Unwise Legislation, 501.

West Virginia State Dental Society, 2.

ORIGINAL PAPERS.

A Comparison of Old and New Style Attachments in Fixed Bridges, Dr. R. B. Howell, 608.

Artificial Enamel, H. L. Belcher, 309.

Consistency, in Terms Applied to Local Remedies, E. T. Loeffler, 336.

Diseases of the Pulp, R. W. Bunting, 411.

Early Care of Children's Teeth with Reference to Prevention of Facial Deformities, M. T. Watson, 390.

Gold Filling with Reference to Plastic Gold, C. G. Morsheimer, 235.

How Dry I Am, George Zederbaum, 522.

Knowing How, D. M. Matteson, 445.

Latest Development in Cast Gold Process, A. L. LeGro, 114.

Mechanical Dentistry Forty Years Ago, E. D. Downs, 229.

Reflex Pains in the Trifacial Nerve, C. J. Lyons, 461.

Selection of a Location for Practice, N. S. Hoff, 344.

Silicious Cements, W. V. B. Ames, 283.

Some Suggestions on Artificial Denture, W. A. Griffin, 5.

Something More and Important about Dr. Taggart, Edmund Noyes, 267.

Strangling Prevented During Extraction, F. H. Skinner, 138.

Syphilis with Special Reference to Recent Experimental Work, H. R. Varney, M. D., 555.

The Nature of Alloys, M. L. Ward, 452.

The Use of Porcelain in Dentistry, C. J. Lyons, 58.

Watch Rochester: Give the Children a Chance, H. Fletcher, 180.

What Can Oral Prophylaxis and Orthodontia do for each other, N. S. Hoff, 19.

REPRINTS.

A Consideration of the Casting Process with Reference to Refractory Metals, M. L. Ward, 503.

Antiseptic Qualities of Saliva, H. C. Ferris, 31.

Bi-Fluoride of Ammonium, Joseph Head, 422.

Cresyl Formo-thymol in Caries of the Third and Fourth Degrees, V. E. Miegerville, 586.

Dental Fees, Brother Bill, 620.

Dental Jurisprudence, C. S. Ayres, 37.

Dentistry and Preventive Hygiene, H. Fletcher, 193.

Extracting Under Nitrous Oxide Anesthesia, A. W. Hall, 534.

Face Beautiful, H. C. Ferris, 42.

Humane Methods and Suggestions for Practice, W. G. Ebersole, 476.

Humane Methods of Wedging, S. E. Davenport, 416.

Indications for the Inlay, J. F. Nyman, 368.

Inlays by the Impression Method, F. T. Van Woert, 528.

Lame Teeth, R. Ottolengui, 260.

Mouth in Relation to Intestinal Diseases, H. G. Longworthy, 364.

New English Anesthetic Legislation, E. C. Kirk, 353.

Oral Hygiene, 29.

Oral Surgery, Wm. Chennery, 255.

Paraform in Treatment of Sensitive Dentine, J. A. Woods, L. D. S., 630.

Pertaining to Pyorrhoea, J. D. Patterson, 470.

Plea for Rationalism in Modern Methods of Practice, C. N. Johnson, 357.

Probable use of Silicate Cements, W. B. Dunning, 312.

Pyorrhoea Prevention and Cure, J. W. Wassoll,

Rational Method of Local Anesthetic, H. Prinz, 242.

Some Practical Points on Taggart Inlays, L. E Custer, A.M. D.D.S., 627.

Therapeutic Committee of British Medical Association, C. N. LeBrock, 371.

Tightening Loose Plates, F. G. Worthley, 467.

Treatment of Interstitial Gingivitis, Eugene S. Tabbot, 138.

INDENTS.

Adapting Wax Inlay Models, 642.

Adhesion Cement, 645.

Ammonium Bifluoride, 220.

Anesthetizing the Pulp, 635.

A Queer Dyspepsia Cure, 645.

Argyrol, 635.

Arsenic Application, 144.

A Systemic Vice, 641.

Banded Crowns, 636.

Bismuth Paste in Treatment of Pyorrhoea, 593.

Boston Conference, 490.

Brushing the Teeth, 598.

Calcium Fluoride in Therapeutics, 544.

Canker Sores, 488.

Cap and Dowel, to fit, 90.

Capping Exposed Pulps, 271.

Care of Mother's Teeth During Pregnancy, 217.

Care Repaid, 217.

Cast Cap and Dowell, 148.

Cast Gold Inlays, 638.

Casting Metal, 431.

Chemical Composition of Teeth, 593.

Chloroform Anesthesia, 95.

Chloroform Fatality, 267.

Chloroform vs Essential Oils as Gutta-Percha Solvents, 216.

Cleveland School Children to be cared for, 640.

Conduction of Filling Materials, 486.

Constructing Abutment for Lateral, 646.

Construction of Crowns, 643 .

Contraction in Inlay Casting, 89.

Cover for Instrument Table, 146.

Cresol, 544.

Curing Pyorrhoea, 275.

Dental Research, 272.

Dentists and Physicians, 641.

Dentistry in Austria, 601.

Dentists for State Institutions, 635.

Dentists Hard Work, 643.

Die Metal,634.

Dissolving Cement from Crown, 637.

Dittmar Cast Shell Crown, 326.

Death from Misadventure, 434.

Decayed Teeth and appendicitis, W. Guy Smith, 134.

Donaldson Broach Suggestion, 147.

Drainage of Abscesses, 636.

Dressing for Putrescent Canal, 646

Drying Root Canals, 638.

Eastern Indiana Dental Association, Officers, 634.

Examination of School Children, 637.

Electro Deposition-of Platinum on Bridge Saddles, etc, 597.

Evan's Museum and Institute, 213.

Eye Sight, 152.

Father of Dentistry, 438.

Flies, 595.

Flies, To Destroy, 635.

Formalin for Sensitive Cavities, 147.

Future Dentistry, 149.

Good Inlay in Fifteen Minutes, 150.

Grinding Edge Tools, 594.

Have a Program, 267.

Heating Casting Instruments, 636.

Hiccough, 265.

Hydrofluoric Acid Burns, 266.

Hygienic Education, 486.

Professor Jessen of Strasburg, 546

Illinois Code of Ethics, 600.

Impression Taking Aids, 46.

Infection, 88.

Instrument for Gold Casting, 640.

Interstate Reciprocity, 637.

Iodoformogen, 644.

Lower First Molar Bridge, 92.

Medical as well as a Dental Course to be Required, 636

Medical Education for Dentists not Desirable, 384.

Mental Science and Dental Practice, 219.

Meeting National Dental Association, 328.

Modification of Anchorage for Regulating Arch, 215.

Moldine, to Soften, 146.

Mounting Detached Post Crown on Cast Base, 94.

Mucin Test, 270.

Museum of the Odontological Section, Royal Society of Medicine, 592.

Neuralgia, 265.

Need New Moulds for Artificial Teeth,, 151.

New Examination for German Dentists, 638.

Nitrous Oxide and Heart Diseases, 380.

Non-Corrosive Instruments, 269.

Occlusion Perfected, 145.

Odorless Deodorant, 634.

Origin of Dentistry, 642.

Oral Hygiene for the Public, 638.

Oral Massage, 634.

Orthoform, 92.

Oral Prophylaxis, 596.

Packing Vulcanite, 97.

Packing Pink Rubber, 148.

Painless Puncture of Alveolar Abscess, 146.

Phenol Gangrene, 383.

Pickling Vulcanite Plates, 591.

Physicians May Not Practice Dentistry, 591.

Platinum Famine Threatened, 431.

Porcelain Crown with Cast Base, 213.

Porcelain Inlay, 381.

Pressure Anesthesia, 265.

Prevention of Cancer, 153.

Professionally Interested, 432.

Proprietary Preparations, 274.

Prosthodontic Criticisms, 98.

Protecting Gold Work from Amalgamation, 634.

Pulp Protection, 639.

Pulpitis Relief, 430.

Quick Method of Making Counter Die, 323.

Quick Method of Replacing a Vulcanite Tooth, 430.

Removing of Amalgam Filings, 144.

Removing of Devitalized Pulp. 145.

Refining Scrap, 147.

Repairing a Hole in Cap Crown, 323.

Report of School Inspector, 435.

Retention of Lower Plates, 325.

Root Banding with Gold, 96.

Root Canal Treatment, 144.

Rubber Dams, 144.

Rules for Eating, 88.

Saliva Antiseptics, 268.

Satin Finish to Aluminum, 641.

School Boys and The Toothbrush, 214.

Separating Medium, 90.

Shield the Innocent, 89.

Silicate Cements, 647.

Silicous Cements, 268.

Silver Salts Applied Safely, 91.

Size of Casting Sprue, 433.

Stability of Copper Alloys, 145.

Stopping Pain, 635

Strength of Cast Gold Crowns, 430.

Supernumerary Teeth, 107.

Swaging Rubber Plates, 47.

Syphilis, 436.

Teaching Children to Masticate Properly, 644.

Temporary Teeth, 324.

Tooth Powders and Washes, 101.

Tongue Blackened by Hydrogen Dioxide, 93.

To Soften Moldine, 639.

Urine Analysis in Pyorrhoea, 324.

Value of Association, 492.

Vulcanite Repairs, 149.

Weighted Lower Denture, 643.

Why the Pulp Canal is so Difficult to Reach, 639.

Zinc Oxide Lining for Inlays, 90.

AUTOGRAPHIC.

Ames, W. V. B., 283, 308.

Arnold, L. H., 431.

Ayres, C. S., 37.

Bardes, D. A., 153.

Belcher, H. L., 309.

Black, G. V., 32, 89, 145, 636.

Bowles, C. H., 83, 271, 308.

Brockaway, A. H., 144.

Brophy, T. W., . 635

Buttrick, T. R., 10. 409,

Bunting, R. W., 305, 411.

Bush, E. M., 430.

Cassidy, J. S., 92.

Chapman, A., 598

Chennery, Wm., 255.

Clark, Mr., 581.

Clapp, G. W., 152.

Cloud, F. E., 146.

Cole, M. 595.

Crouse, J. N., 433.

Custer, L. E., 627.

Davenport, S. E., 416.

Dick, Wm., 89.

Donohower, A. F., 634.

Downs, E. D., 229.

Dumas, A. W., 131.

Dunning, W. B., 312, 426.

Ebersole, W. G., 476.

Fay, M. L., 46.

Ferris, H. C., 31, 37, 42.

Flanagan, A. J., 92, 149.

Fletcher, H., 88, 180, 193, ,644.

Fuller, E. S., 266.

Giffin, W. A., 5, 16.

Gilmer, T. L., 490.

Green, J. W., 150.

Guilford, S. H., 93, 647.

Hall, A. W., 534.

Hall, J. S., 405.

Hall, L. P., 295.

Harris, C. C., 486.

Harrison, A. M., 91.

Haskell, Dr. 643.

Haskell, L. P., 101.

Hayes, G. B., 145.

Head, Joseph, 223, 422, 427

Hoff, N. S., 19, 344, 450, 496, 571. 605, 607.

Howell, R. B., 608.

Hutchinson, R. G. Jr., 425

Hutchinson, Wood, 154.

Johnson, C. N., 278, 357.

Kelsey, H. E., 216.

King, O. U., 268.

Kirk, E. C., 35, 353. 641

Kousleff, C., 488.

Land, C. H., 13, 306, 407.

Lane, J. G., 95, 636., 640.

Latcham, H. E., 634.

Longworthy, H. G., 364.

Laupe, A. A., 399, 581.

LeBrocq, C. N., 371.

Le Cron, 432, 434.

Le Gro, A. L., 114, 133.

LeRoy, F. B., 34.

Leger-Dorenz, H., 591.

Linet, E., 49.

Lodge, E. B., 544, 638.

Loeffler, E. T., 336.

Lyons, C. J., 58, 82, 149, 448, 451, 461.

Marion, G. P., 147.

Mathews, Wm. 266.

Matteson, D. M., 445.

McCallin, Sidney, 597.

McCann, T. A., 64 .

McDonald, F. W., 79, 302.

McManus, C., 642.

Merritt, Dr., 425.

Michaels, J. P., 34.

Miegerville, V. E., 586.

Miller, H. S., 98.

Morsheimer, C. G., 235.

Muir, R. M., 124, 128, 132.

E. Noyes, 207.

Nyman, J. F., 368.

Oakman, C. H., 78, 84, 570, 578.

Ottolengui, R., 220, 260.

Patterson, J. D., 470.

Perry, Dr., 269.

Price, W. A., 89.

Prinz, H., 242.

Prothero, J. H., 643.

Pusey, W. A., 438.

Raymond, H. C., 76.

Roach, F. E., . 646

Read, Thomas, 600.

Reed, W. D., 449.

Reed, W. L., 637.

Rettger, L. F., 33.

Rich, Celia, 90.

Richards, W. M., 324.

Sawyer, A. J., 97.

Schlegel, G. S., 214.

Schoenbrod, A. M., . 646

Skinner, F. H., 636.

Skinner, F. N., 138.

Smith, F. M., 144.

Smith, W. Guy, 134.

Squires, G. B., 104.

Talbot, E. S., 138.

Temant, E. W., 144, 635.

Thiersch, W., 598.

Thomas, J. D., 381.

Thorpe, B. L., 638.

Trueman, W. H., 216, 268, 634.

Thompson, 73, 81. 127, 581.

Tuller, R. B., 148, 265, 274, 328.

Tweedle, W. H., 430.

Ulsover, E. S., 642.

Varney, H. R. M. D., 555, 581.

Van Woert, F. T., 528.

Voyles, S. H., 383, 638.

Viall, E., €45.

Ward, M. L., 129, 302, 449, 452, 503

Wassall, J. M., 238.

Watkins, S. G., 217.

Walton, S. G., 325.

Waltz, A. S., 323.

Watson, M. T., 390, 410.

Weeks, F. T., 639

Weeks, Thos., E. 272.

Weisse, F. D., 32.

White, O .W., 406.

Whitman, E. L., 449.

Wood, C. P., 408.

Woodbury, Wm. R., 492.

Woodle, J. M., 644.

Woods, J. A., 630.

Worthley, F. G., 467.

Young, D. H., 326.

Zeberbaum, Geo., 522.

ANNOUNCEMENTS.

Alumni Association University of California, 278.

Angle's Patent Decision, 649.

Australian Dental Congress, 49.

Banquet to Dr. Butler, 50, 225.

C. R. Butler, Banquetted, 106.

California State Society, 223, 331.

Chicago Odontographic Society, 108.

Correction, 105.

Dental Index Bureau, 332.

Dental Pedestrian Owre, 105.

Eastern Kentucky Dental Association, 155.

Florida State Association, 225.

Dr. C. A. Hawley Removed to Washington, D. C., 49.

G. V. Black Banquet, 649.

Harvard's New Building, 650.

Illinois Board Examination, 548.

Illinois State Dental Society, 104, 155, 224, 548.

Indiana State Board of Examiners, 650

Indiana State Dental Association, 332.

Institute of Dental Pedagogics, 648.

International Dental Exhbition, 278.

Interstate Dental Fraternity, 106, 155.

International Dental Congress, 156.

Iowa rBoad of Examiners, 279.

Iowa State Board of Examiners, 649.

Kentucky Dental Association, 155, 224.

Lake Erie Dental Association, 548.

Marquette Dental Society, 107.

Membership National Dental Association, 50.

Michigan First District Annual Clinic, 106, 440, 603.

Michigan State Society, 223, 331, 603.

Missouri State Association, 156, 442, 548.

E. C. Moore Retires from Practice, 278.

National Dental Association, 279.

New Jersey Dental Society, 104.

North Ohio Association, 161, 549.

New York Odontological Society, 550.

New York Seventh District Society, 160.

Ohio State Dental Society Officers, 51.

Ohio State Dental Society, 550, 604.

Ohio Board of Examiners, 224 496, 549.

Pennsylvania Board of Examiners, 224.

Pyorrhoea, 442.

Ransom & Randolph Co., 107, 279.

Reorganization Michigan Dental Society, 49.

St. Louis Society of Dental Science, 104, 105,

S. W. Michigan Dental Association, 496.

Tennessee Association, 225.

Virginia State Dental Association, 156.

West Virginia Dental Society, 50.

Xi Psi Fraternity, 51.

BIBLIOGRAPHY.

Abbott's Bacteriology, 542.

Dental Metallurgy, 440.

Gorgas Dental Medicine, 441.

Gray's Anatomy, 86.

Practical Dentistry, I. N. Broomell, 86.

Johnson's Operative Dentistry, 52.

Long's Materia Medica, 543.

Prinz's Book on Therapeutics, 332.

Simons New Chemistry, 540.

OBITUARIES.

W. H. Dorrance, 84.

Dr. Luis L. Dunbar, 142.

Alison W. Harlan, 263.

C. N. Pierce, 379.

F. S. Whitslar, 539.

IN MEMORIAM.

Dr. Joseph W. Wassall, 633.

the tin foil is preserved, the plate will surpass a metal plate as well as an ordinary rubber plate. The disagreeable sensation of heat is done away with by this rubber plate with a metallic base, the food particles do not stick as firmly as they would to a rubber plate, and the elasticity of the latter, which is one of its advantages over a metal plate, is preserved.—Dr. E. Linet in Dec. 1908 *Cosmos*.

### ANNOUNCEMENTS.

Reorganization of Michigan Dental Societies.—The reorganization of the several local societies, of Michigan, into component parts of the State Society has made such progress that but two more districts will probably be organized in the near future. It seems impracticable to get the dentists of the Northern Peninsula to think they can sustain a dental society, and that part of the state will have to go for the present. Beginning with 1909, the *Dental Summary* will print the proceedings of the state and district society, and it is planned to have the reports as complete as is possible.

Dr. Charles A. Hawley, has removed from Columbus, Ohio, to Washington, D. C. and will engage in the practice of Orthodontia, as a specialty, at 815 Connecticut Ave.

The Second Australian Dental Congress.—The dentists of Victoria have decided to hold a great dental Congress in Melbourne, Australia, October 25th to 30th,

1909. All dentists practicing in Australia and New Zealand will cooperate and invitations are being extended to practitioners of other countries. The program outlined promises a great meeting.

---

WEST VIRGINIA DENTAL SOCIETY.—This society will meet in Wheeling next year, October 13th to 15th, and the dentists of Wheeling have printed a very fine appointment book with a reminder on every page of the time or place or value of the meeting and some urgent reason for attending. It is the most unique and aggressive campaign document we have ever seen. It ought to produce results.

---

MEMBERSHIP IN THE NATIONAL DENTAL ASSOCIATION..—At the 1908 meeting, the National Dental Association adopted an amendment making all members in good standing in their State Dental Societies, or their Allied Societies, eligible to membership in this Association, by presenting to the proper authorities at the regular meeting a certificate signed by the president and Secretary of any such Society.

Those desiring to take advantage of their privileges under said Amendment should act promptly, as the National Association meets early this year, the last Tuesday of March 1909, at Birmingham, Ala.

Blanks can be secured from the secretaries of the various State Dental Societies or the undersigned.

H. C. BROWN, *Cor. Sec'y.*,
185 East State Street, Columbus, O.

---

COMPLIMENTARY BANQUET.—The dental profession of Cleveland, Ohio, will give a complimentary dinner to one of its most honored members, Dr. C. R. Butler, on March 11th, 1909, at seven o'clock P. M., in commemoration of the completion of fifty one years of dental practice by the Doctor, This will be a democratic affair to which all ethical dentists are invited. The price per plate will be within the reach of all. Those desiring a plate at the banquet will kindly notify the secretary at least ten days before.

DR. S. B. DEWEY, *Secretary*,
Lennox Building, Cleveland, O.

---

THE NEW YORK ALUMNI ASSOCIATION of the XI PSI PHI FRATERNITY met at the St. Denis Hotel on Nov. 18th, and elected their officers for the ensuing year. It was decided to hold a banquet on January 30th, 1909.

Our membership has passed the 200 mark and it is earnestly desired that every Alumnus be present.

To any who have not received full particulars the same will be gladly furnished by our Secretary.

J. N. GELSON,
673 Vanderbilt Ave.,
Brooklyn, N. Y.

---

DEAR DOCTOR.—At the forty-third annual meeting of the Ohio State Dental Society held in Columbus, December 1st, 2nd and 3rd, 1908, the following officers were elected: President, W. H, Whitslar, Cleveland; 1st V. President, M. H. Fletcher, Cincinnati; 2nd V. President, A. O. Ross, Columbus; Secretary, F. R. Chapman, Columbus; Treasurer, W. A. Price, Cleveland. Directors for three years; L. P. Bethel, Columbus; C. I. Keely, Hamilton; J. R. Callihan, Cincinnati; Henry Barnes, Cleveland.

Yours truly,
F. R. CHAPMAN, *Sec'y*.

Text Book of Operative Dentistry.—This is a new book of 750 pages by various authors,—sixteen in number,—under the editorial supervision of Dr. C. N. Johnson. The work does not pretend to be a treatise on the subject of Operative Dentistry, but is a compilation of the matured views and practices of the several authors on the different phases of that work. It is somewhat cyclopedic in character and yet lacks the balance and symmetry essential to the treatment of a subject in a cyclopedic manner. In illustration of our meaning we would cite the fact that nearly one half of the space is given over to a scientific exposition of Dental Anatomy and Technical Orthodontia neither of which to-day are taught in our schools as operative subjects, nor practiced by the profession as such, at the same time oral prophylaxis, the basis if not the essential of operative treatment is scarcely referred to except in an article on oral hygiene and another on the treatment of Pyorrhoea Alveolaris; and the essentially practical item of cavity preparation and principles of adopting the various filling material receives from eight or nine contributors less than two hundred pages. We do not wish to be understood as undervaluing the quality of the material contributed, for it is for the most part ideal and represents modern practice perfectly, and while it may comply with all the requirements of the average practitioner and teacher, the space devoted to the various text subjects in a work intended to exemplify modern operative practice, as the preface sets out, is, it seems to us, altogether out of proportion. The use of such books in the class room will tend to produce unsymmetrical training unless the instructor is wise and strong enough to put a more equal and proportionate emphasis on all subjects properly taught under his classification. As a work for a practitioner it is much more valuable and it will find a welcome place in the library of every progressive dentist. We most heartily wish that dentists would buy and read such books. It would be ideal if each of the men who has contributed to this book would spend the time to place the various subjects he has treated before us in his own individual capacities as author and editor. Perhaps we have not yet reached the time when we can produce books on professional topics the profession will not buy in sufficient quantities to justify their publication without reference to the student in college, but we shall need the stimulus of such contributions to our literature to develop professional culture and keep us from being absorbed in and possibly dwarfed by technical details. We do not criticise the article on Dental Anatomy for it is written with that care and scientific knowledge that characterize everything done by Doctor Turner; neither do we object to the most valuable contribution of Doctor Pullen on Orthodontic science and Technique; he is a master of his art and has made a most valuable contribution to his subject. We should be most happy to see these articles printed in volumes by themselves, for we believe they would receive the appreciation they clearly deserve. Then again in this work we regret that Dr. Johnson has not put more of his own work into the text, not that we don't appreciate Webster, Owre, Capon, Nyman, and all the others, but we expected Johnson and are disappointed to find that he exhausted his efforts on mere editorial work. We could have edited a book of this sort ourselves, but we couldn't write such a book on operative dentistry as Doctor Johnson, and now that he has allowed himself to be side tracked as a mere editor we fear the profession will lose forever the great fund of practical and scientific knowledge his wide experience and unusual opportunities have brought to him, and which no one has a better faculty for presenting to the profession in a literary form that would attract readers and help to educate a most reluctant profession to appreciate its literature, which is the one thing that will endure and give us a place in the world's history. Now this is a queer book review, but it expresses our sentiment, which may be wrong or biased, but it is honest. The gentlemen who have contributed to this work have all done their parts well, and the publishers have made us a handsome book, and we are really delighted to have it and shall keep it on our desk for constant reference until we have completely absorbed and digested its contents, and then perhaps we shall be better able to appreciate the arduous labor of love on the part of our contributors. And we trust that every practitioner in the land may at once buy and read this book before it gets out of date, for the editor tells us that one chapter had to be rewritten after it had been put into type because of a new discovery which made the technical procedure described in that chapter a back number. It is therefore necessary for you to have this book which is up to date as you probably are not. It is published by P. Blakiston's Son & Co., Philadelphia, and cost six dollars, but it is worth many times its cost.

